# Use of Population Weighted Density Index for Coronavirus Spread in the United States

**DOI:** 10.36469/001c.117784

**Published:** 2024-07-17

**Authors:** Huseyin Yuce, Hannah Stauss, Adrienne Persad

**Affiliations:** 1 Department of Mathematics New York City College of Technology; 2 Department of Computer Systems New York City College of Technology

**Keywords:** population, mortality, density, population-weighted density, COVID-19

## Abstract

**Background:** Understanding how population density affected the transmission of COVID-19 is vitally important, since crowded cities were the epicenters for the disease. Since human contact was the main cause of the spread, population-weighted densities have been shown to be a better measure than conventional densities, since the variation in density across subareas matters more than the density in the total area.

**Objectives:** This study investigates the impact of population-weighted density and other demographics on the rate of COVID-19 spread in the United States.

**Methods:** The study considers population-weighted density and many other demographics. The population-weighted density index is the weighted average of density across the tracts, where tracts are weighted by population. Multivariate analysis has been used to determine the elasticity of the spread.

**Results:** Using U.S. county-level data, we calculated the elasticity of COVID-19 spread with respect to population-weighted density to be 0.085 after controlling for other factors. In addition to the density, the proportion of people over 65 years of age, the number of total healthcare workers, and average temperature in each county positively contributed to the case numbers, while education level and income per capita had a negative effect.

**Discussion:** For the spread, understanding the population characteristics and dynamics is as important as understanding the infectious disease itself. This will help policy makers to utilize and reallocate the resources more effectively. If the spread is successfully contained early, there will be less stress placed upon the healthcare system, resulting in better healthcare access for those who are sick.

**Conclusions:** Our analysis suggests that population-weighted density can be a useful tool to control and manage outbreaks, especially within the early stage of the spread. We presented the early dynamics of the spread and recommended a policy measure on how to transfer healthcare workers from low-spread-risk areas to high-spread-risk areas to utilize resources better.

## INTRODUCTION

The COVID-19 pandemic posed a unique challenge to public health officials around the world since its start in late 2019. One key question was how much population density contributed to the spread of SARS-CoV-2 as the pandemic progressed.^1^ It became clear that population density played a critical role in determining the severity of the outbreak.^1–6^ The impact of population density on COVID-19 spread was shown by Baser in Turkish data^1^ and by Carozzi in U.S. data.^7^ In this study, we show that the spread leads to an initially positive and significant relationship between the impact of COVID-19 and population-weighted density at the county level.

There have been numerous articles and reports about the COVID-19 pandemic since its start. Martins-Filho considered a group-level analysis for the Brazilian data.^8^ Wong and Li considered the raw population density as its explanatory variable in the U.S. county level.^9^ Sy et al considered population density, percent of public transportation, percent of private transportation, and median household income as its explanatory variables at the U.S. county level.^5^ Smith et al explored the association between environmental covariates and COVID-19 transmission intensity.^10^ A study from Johns Hopkins University concluded that a higher county population, a higher proportion of people age 60 and over, a lower proportion of college-educated people, and a higher proportion of African Americans were all associated with greater infection and mortality rates.^11^ Lima et al modeled the COVID-19 cases and related deaths with explanatory variables: walk score, population density, population size, and the number of days under a stay-at-home order.^12^ Hazarie et al considered 163 cities chosen from 4 different continents and reported a global trend based on human mobility.^4^

Density is one of the most fundamental characteristics of an urban area.^13^ However, raw population density, simply population divided by county area, is not a good measure of the density at which the population actually live.^2^ Human contact is the main cause of the spread of infectious diseases. Therefore, population-weighted densities are a better measure than conventional densities, because the variation in density across the subareas matters more than the density in total area.

The goal of this article was to derive population-weighted density and COVID-19 spread for the major counties in the United States. Then, using the data points, the relationship with the density and the spread of coronavirus was analyzed in those counties, controlling for education level, wealth, healthcare force, temperature, and demographics. We computed the elasticity (the percentage change in the spread for a 1% change in population-weighted density, holding all other independent variables constant as a measure of sensitivity) of the COVID-19 spread and compared elasticities over time. We suggest that population-weighted density can also inform us on how to mobilize each region’s healthcare force from low-spread-risk areas to high-spread-risk areas to utilize the resources better.

## METHODS

### Data

The dissemination of information regarding the transmission of COVID-19 in the United States. has relied heavily on data provided by sources such as the U.S. Census Bureau (https://www.census.gov), Johns Hopkins University (https://coronavirus.jhu.edu), and state and local governments. However, certain irregularities within specific counties have been discovered. Notably, cumulative COVID-19 cases experienced significant declines on various dates for several counties. At times, these unexplained reductions were by as many as 500 000 cases in a single county (eg, Cook County, Ill.). Moreover, the ultimate cumulative case total for a number of these counties failed to align with figures reported by other sources. We opted to utilize the Johns Hopkins dataset as the primary source, supplementing it with missing values from USAFacts (https://usafacts.org) and cross-verifying the results against state-level sources.

Consequently, our confirmed cases and mortality datasets ultimately present the most accurate depiction of COVID-19 at the county level between January 22, 2020, and March 9, 2023. We compiled a data set that combines information on COVID-19 cases and deaths, population-weighted density, temperature, healthcare resources, and various demographics at the county or equivalent level. The U.S. Census Bureau provided healthcare and demographic data, including gender distribution, median personal income, the percentage of people with a bachelor’s degree or higher, and the number of people older than 65 in each county. We obtained the average yearly temperature for each county from https://weatherspark.com. Dr. Devin Michelle Bunten’s work at the Massachusetts Institute of Technology (https://www. devinbunten.com/data) provided the measure for weighted density. Whenever available, we used current data for the year 2021. However, there were exceptions. We used the average temperature calculated as a 100-year average from 1901 to 2000. The education level was measured as an average for 2017-2021, and the weighted density was calculated using data from 2012-2016.

### Population-Weighted Density and Correlation

Before New York City placed restrictions on its residents to combat disease spread, the number of cases in New York City was close to 20 times the number of cases in Los Angeles County.^14^ This difference can be explained by the population-weighted density. **Table 1** shows the rankings among the weighted density index, plain density, and population.

**Table 1.** Top 20 Counties According to High Population Weighted Density

**State**	**County**	**Weighted Density**	**Ranking Weighted Density**	**Population**	**Ranking Population**	**Population Density**	**Ranking Population Density**
NY	New York	137842.79	1	1634989	20	72167.6	1
NY	Bronx	91472.48	2	1436785	26	34167.97	3
NY	Kings	64874.7	3	2606852	8	37340.68	2
NY	Queens	51945.12	4	2310011	11	21237.95	4
NJ	Hudson	40391.33	5	668526	95	14454.64	6
CA	San Francisco	33631.33	6	850282	65	18131.88	5
MA	Suffolk	29072.29	7	767719	78	13179.87	7
PA	Philadelphia	24544.71	8	1559938	23	11625.88	8
DC	District of Columbia	22162.93	9	659009	100	10777.79	9
VA	Arlington	20157.44	10	226092	285	8698.59	11
VA	Alexandria	18547.23	11	151473	425	10065.07	10
NY	Richmond	17717.78	12	473324	144	8136.07	12
NJ	Essex	16765.98	13	792586	75	6284.49	15
IL	Cook	15915.99	14	5227575	2	5531.88	17
NJ	Passaic	15665.89	15	507204	134	2726.84	42
CA	Los Angeles	15225.24	16	10057155	1	2478.24	49
MD	Baltimore City	15224.8	17	621000	107	7671.99	13
HI	Honolulu	15121.15	18	986999	46	1643.34	87
FL	Miami-Dade	13170.97	19	2664418	7	1403.31	105
CA	Alameda	12197.36	20	1605217	21	2171.87	59

Los Angeles County ranks first in population (and 49th in raw population density), and its rank in weighted density is 16th, as opposed to New York, which ranks first in weighted density index and 20th in population. Los Angeles is 6 times more populated than New York but is hemmed in by mountains, limiting how far the commuting zone can reach, and is spread out more than New York. However, according to population-weighted density, an average New Yorker lives in a census tract with more than 137 000 people within a square mile, 9 times more than the weighted density of Los Angeles County (**Table 1**).

The density of the urban area *D* is the total population *P* divided by the total area *A*, *D=P/A*. Let $p_{i}$ be the population and $a_{i}$ be the subarea of the total area, by definition $P=\sum_{i}p_{i}$ and $A=\sum_{i}a_{i}$. Therefore, the density for each subarea is $d_{i}=\frac{p_{i}}{a_{i}}$.

Population-weighted density $D_{p}$ is the mean of the subarea densities weighted by the population of the subareas:

(1)Dp=1P∑ipidi

The difference between population-weighted density and conventional density is a simple function of the variance in density across the census subareas and conventional density.^15^ The differences then will depend on the variation in density across the subareas shown by Craig.^2^ In the United States, this measure has been part of the national statistics since 2010.^1^

**Figure 1.**
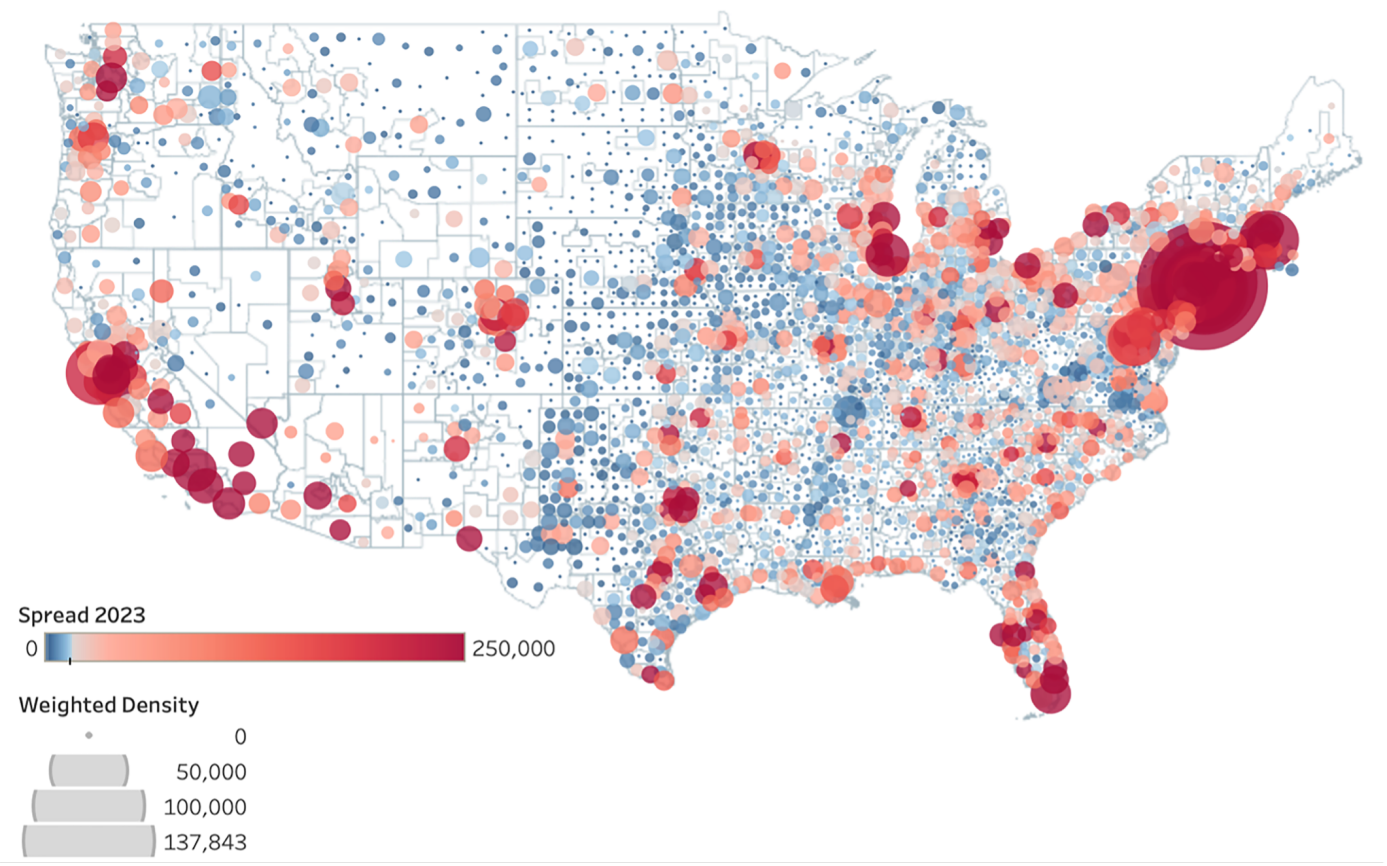
COVID-19 Spread vs Population-Weighted-Density Map by County

The map in **Figure 1** shows COVID-19 spread and population-weighted density. It clearly indicates that denser locations (bigger bubbles) also have higher spread of the disease (redder locations). There is a relatively strong positive relationship between population-weighted density and the spread of disease. Correlation coefficient was calculated as 0.79825 at *p* < .0001. The correlations among the spread, mortality, population-weighted density, number of healthcare workers, and number of people over 65 years old are the strongest. There is strong positive correlation between the spread of the disease and the mortality due to COVID-19, 0.95 (*p* < .0001); the number of healthcare workers, 0.92 (*p* < .0001); the number people over 65 years of age, 0.96 (*p* < .0001).

**Figure 2.**
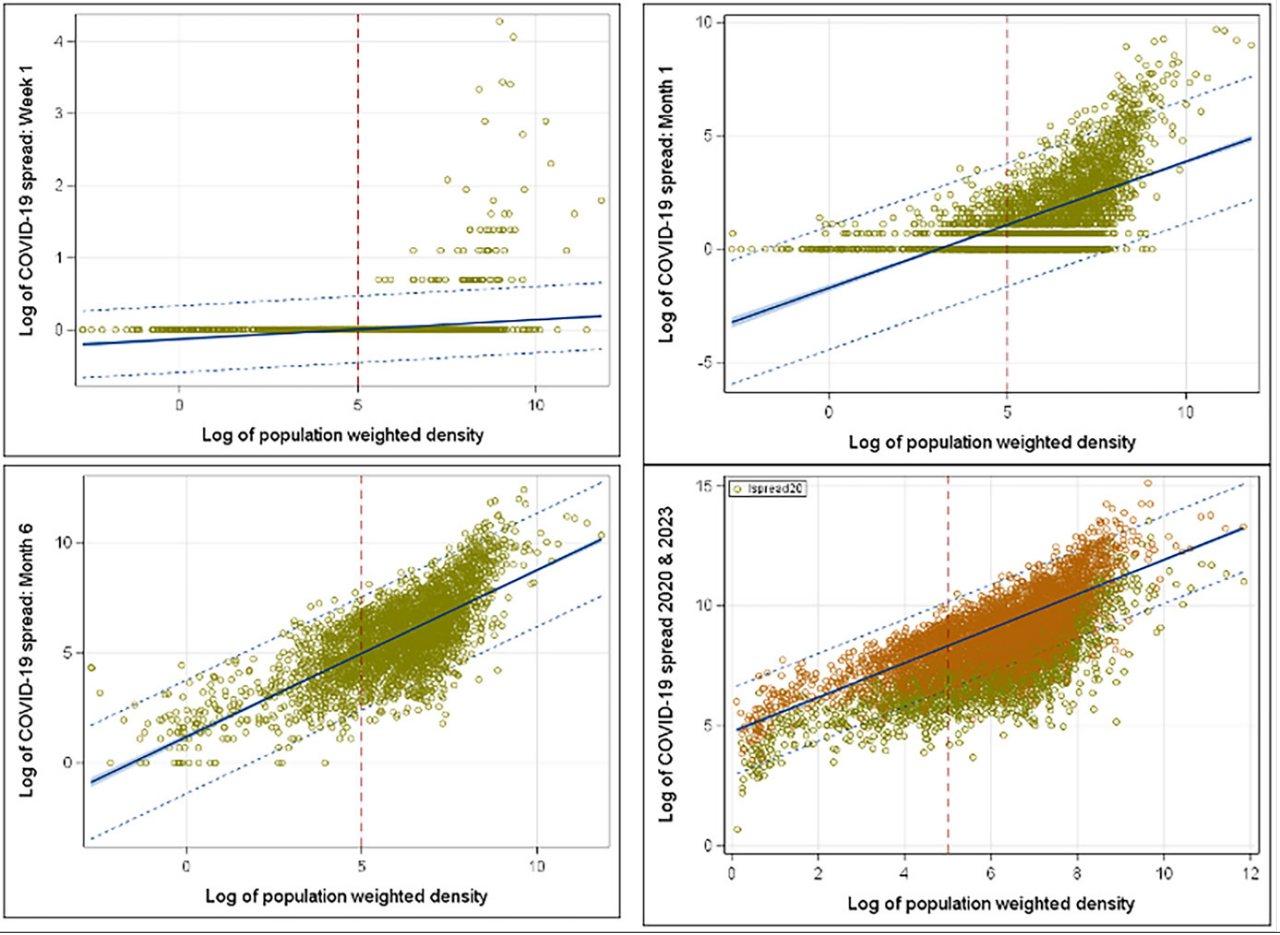
COVID-19 Spread Trend: 2020-2023

To understand COVID spread based on the population-weighted density, the early stage of the spread provides better understanding and visualization without the clutter of the full data set. Thus, we looked at the year 2020 with week 1, month 1 (March 2020), month 3 (May 2020), and month 6 (November 2020) starting from March 1, 2020. Within the first week, all COVID cases were in the counties with high-weighted density in the population (week 1 in **Figure 2**). At the end of the first month, it started to spread over the less dense counties (month 1 in **Figure 2**). **Figure 2** shows how COVID spread rose within the dense counties rather quickly and looked like the spread shape of 2023 within 6 months (month 6 in **Figure 2**). Policy makers need to advance a plan to contain the disease within 6 months and try to contain the spread within the identified dense counties.

### Model Selection and Multivariate Regression

In addition to population-weighted density, risk factors considered include education, wealth of each county, number of healthcare workers, proportion of females to males in the population, and people 65 years of age or older. To determine the relationship between these variables and the spread, we considered the multivariate linear model,

(2)yi=β0+β1x1i+⋯+βkxki+ϵi

where *i* = 1,2,…,*m* are counties and $\epsilon_{i}$ is the error term (assuming *k* variables). The coefficient matrix β can be obtained by regression analysis. Then the model in matrix notation is, Y=Xβ+ϵ, where $\beta^{\prime }=\left(\beta_{0},\beta_{1},\ldots ,\beta_{k}\right)$ is the parameter vector. The vector of least-squares estimates, $\hat{\beta^{\prime }}=\left(\hat{\beta_{0}},\hat{\beta_{1}},\ldots ,\hat{\beta_{k}}\right)$, was obtained by solving the set of linear equations,

(3)β^=(X′X)−1X′Y

provided that ***X**′**X*** has full rank. The matrix *C* = (***X***′***X)***^-1^ provided the variances and covariances of the regression parameter estimates $V\left(\hat{\beta }\right)=\sigma^{2}C$, where $\sigma^{2}=V\left(\epsilon_{j}\right).$ The variables such as population-weighted density, total healthcare workers, senior citizens, education level, and county’s per capita income in logarithmic form allowed us to measure elasticity.

For the model selection process, the variables with the higher correlation coefficients were chosen first and progressively searched for the higher value with the minimal Mallows’ number $C_{p}$. Mallows’ $C_{p}$ was calculated as:

$$C_{p}=\frac{RSS_{p}}{S^{2}}–N+2(p+1),$$

where $RSS_{p}$ is the residual sum of squares for a model with *p* predictor variables, *S^2^* is the residual mean square for the model (estimated by MSE) *N* is the sample size, and *p* is the number of predictor variables. Since, *C_7_* = 8, the model was unbiased, and *R^2^* = 0.9588 indicated that the 95.88% of the variation can be explained by this model.

**Table 2** is the COVID-19 spread model with 7 explanatory variables. The variance inflation factors were mostly small (<5) with the exceptions of older adults and healthcare workers (>5), indicating correlation between these variables and the predictor variable spread. The residuals of the model were approximately normally distributed, and there was no heteroscasticity in the model. The model predicted about 96% of the variation within the data.

**Table 2.** Parameter Estimation of COVID Spread Model in the United States

**Variable**	**Parameter Estimate**	**Standard Error**	***p* > |*t*|**	**95% Confidence Limits**		**VIF**
Intercept	1.84684	0.34757	<.0001	1.16535	2.52833	0
Log of population-weighted density	0.08518	0.00566	<.0001	0.07408	0.09629	2.99056
Log of older adults (age ≥65 years)	0.83407	0.01050	<.0001	0.81348	0.85465	6.96596
Log of total healthcare workers	0.13220	0.00776	<.0001	0.11699	0.14741	7.79970
Log of income per capita	-0.06620	0.03395	.0513	-0.13277	0.00037	1.78456
Log of education	-0.16869	0.02283	<.0001	-0.21345	-0.12393	2.41899
Temperature	0.00815	0.00069	<.0001	0.00679	0.00952	1.28697
Female/male proportion	-0.00638	0.00075	<.0001	-0.00787	-0.00490	1.18671

Abbreviation: VIF, variance inflation factor.

### Selected State Comparisons

As indicated in the map (**Figure 1**), denser locations also had higher spread of the disease. In the state-level analysis, we considered Arizona (AZ), California (CA), Florida (FL), Illinois (IL), New York (NY), and Texas (TX). Selection criteria for the states are based on the high-spread, high-population-weighted-density counties which include New York (NY), Los Angeles (CA), Maricopa (AZ), Cook (IL), Miami-Dade (FL), and Dallas (TX).

**Figure 3.**
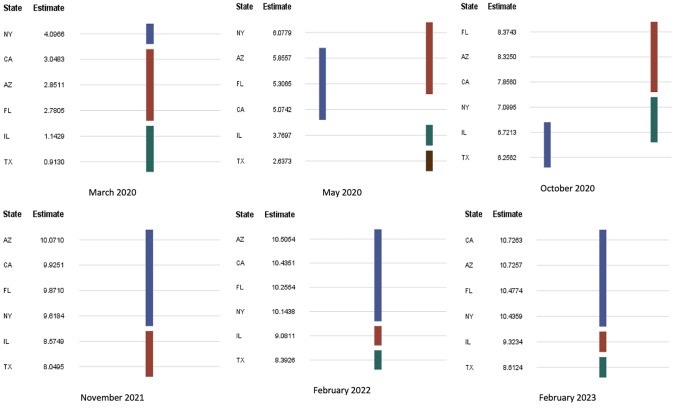
Duncan Test on COVID-19 Spread Over Time

While there were differences in mean COVID-19 spread between states, it is interesting to see how they grouped in time. Duncan’s test was done with significance level to identify the groupings of the states. **Figure 3** shows the groupings on the benchmark dates. In March 2020, NY had the highest spread with its own group while the mean spread differences in CA, AZ, and FL were insignificant, forming the second group. As COVID restrictions were implemented and the population learned more about the disease, NY COVID spread decreased and formed a new group with IL and TX on October 2020 as CA, AZ, and FL mean spreads rose. As we learned how to manage the disease, the spread curve flattened, and groupings settled as shown in subfigures February 2022 and February 2023 in **Figure 3**. Mean comparisons of the COVID-19 spread by state can give policy makers reasonable indications of what rules to put forward and how effective these rules are. Also, through these comparisons and groupings, officials can identify states in urgent need of action. For example, NY restrictions resulted in a significant decrease, putting the state in line with IL and TX levels. The rate at which the disease spreads is measured by its elasticity. The trend of elasticity over time can be used to observe the effectiveness of COVID-19 restriction policies.

Early COVID-19 spread depended highly on population-weighted density. However, weighted density lost its importance as we learned how to deal with the disease and gain access to the healthcare resources within dense metropolitan cities. Therefore, the COVID-19 spread model for NY and CA (as well as others) flattened over time (constant elasticity with almost no vertical shift). For example, COVID spread within the first 10 months resulted in high dependence on the weighted density, while the spread in April 2023 did not. Linear multivariate regression, analysis of variance comparisons, correlations, and visualizations were performed using SAS (v. 9.4), Python, and Tableau.

## RESULTS

There were significant associations between socioeconomic factors and the growth of the spread. Counties with higher education levels had significantly lower COVID-19 infection rates. Counties with a 10% higher population with an education level of at least a 4-year college degree were associated with 1.6% lower disease spread. This trend can be explained by the fact that people with higher education levels have a better understanding of the virus and take restrictions more seriously. They are also more likely to be able to work from home. This result is consistent with previous studies.^1,16^

The population of older adults (≥ 65 years) was strongly associated with the growth rate of the spread. Each percentage point in the senior population results in an 83% increase in the spread growth rate. This may be because people aged 65 years and older generally have weaker immune systems than the rest of the population.^17^ Older adults were the biggest contributing factor to our model. Heavily dense counties have more seniors, and mortality is greater among them. The overall shape of spread among older adults was similar to the national spread (COVID spread 2023, **Figure 2)**, explaining the 97% correlation.

Since the higher number of healthcare workers in metropolitan counties was related to a higher rate of testing, there was a positive correlation between total number of healthcare workers and the spread of the disease. A 10% increase in healthcare workers resulted in a 1.3% higher disease spread in the county. These findings are consistent with previous U.S. studies that found statewide testing is the most significant predictor of the county infection rate.^11^

Income per capita was inversely related with the spread of the disease, consistent with the literature. For every 10% increase in income per capita, the spread decreased by about 0.63%. This trend can be explained by the fact that having a high income could indicate a better ability to effectively self-isolate, access to better healthcare, or jobs that can easily be transitioned to working from home. Once the disease enters low-income neighborhoods (or counties), it becomes very difficult to control the further spread of infections, as low-income households may have neither the resources to effectively self-isolate nor jobs that can easily be switched to work-from-home. It is therefore not surprising that the number of cases started to increase dramatically in low-income areas.^18^

The proportion of female to male population was inversely associated with growth rate of the spread (albeit not strongly). Counties with a higher female/male population ratio had a lower rate of cases. An increase of 1 female per 100 males in a county resulted in a decline in the spread by a factor of 0.99364 (*e^-^*^0.00638^) (ie, counties with a 1% higher female/male proportion were associated with 0.6% (*e^-^*^0.00638^) lower disease spread). A recent study showed that men and women have the same prevalence of COVID-19, although men who have tested positive for COVID-19 were at higher risk for worse outcomes and death, independent of age.^19^

Temperature had a positive effect on the COVID spread in the U.S. counties with 1° F higher temperature resulting a spread factor of 1.008 (*e*^0.00815^) (ie, counties with 10% higher temperature were associated with 0.8% higher disease spread, which is not supported the current literature.^1,20^ However, more than half of the U.S. population, 62.68%, live in the states that are about 10 degrees warmer than the national average of 54.05° F (**Supplemental Online Material**, **Table S3**). Hence, the tendency of warmer states to have higher spread is consistent with the model, since most of the population was in the warmer states.

The rate of COVID-19 spread was infamously exponential at the early stages of the pandemic (**Figure 2**). However, classic epidemiological models suggested that the rate of growth itself depends on the number of incidences, and the rate declines as the number of incidences grow. The idea of slowing the spread so that fewer people need to seek treatment at any given time is known as “flattening the curve.” The “curve” refers to the projected number of people who will encounter COVID-19 over a period of time. The faster the infection curve rises, the faster the local healthcare system becomes overloaded beyond its capacity to treat people.

Performance of any healthcare system depends on the availability of a sufficient number of skilled healthcare workers. Additionally, it is crucial for these health workers to be mobile, as areas with high population-weighted density are often the first to experience a surge in COVID-19 cases. For example, in the first months of the COVID-19 epidemic, healthcare workers flew from San Francisco to New York City, where there was a higher need for services.^21^ Also, the Navy hospital ship U.S.N.S. *Comfort* and the Defense Department ran an alternate care facility at the Jacob K. Javits Convention Center in New York City. The vessel housed up to 2500 patients, 500 beds, and 96 ICU beds.^22^ Availability of such mobility can be directed to areas with the high population-weighted density. As for channeling healthcare resources effectively, possible transfer of healthcare workers can be scheduled from low population-weighted-density counties with relatively high numbers of healthcare workers to high population-weighted density counties with relative low numbers of healthcare workers.

**Figure 4.**
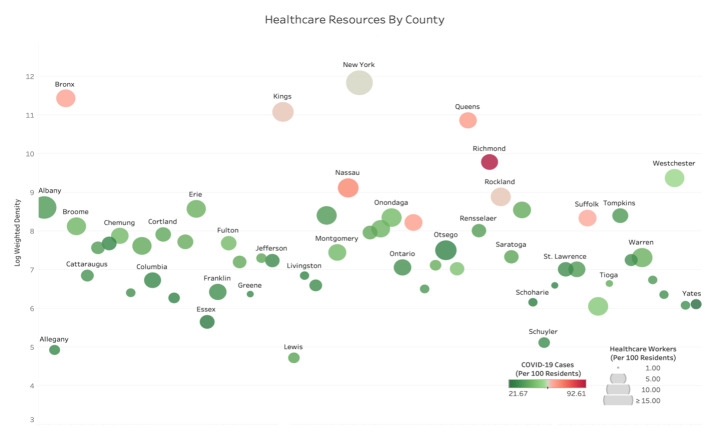
Possible Transfer of Healthcare Workers Among Counties in New York

As a state-level example, consider NY. **Figure 4** shows density, spread, and healthcare workers (as a resource to fight the virus). The intensity of green and red shades shows the excess of healthcare workers and the high intensity of the spread, respectively. The high population-weighted density is indicated by the larger bubbles. In **Figure 4**, large, green counties like Franklin, Columbia, and Otsego have low population-weighted density with a relatively high number of healthcare workers, and the small red counties like Richmond, Bronx, and Queens have high population-weighted density with a relatively low number of healthcare workers. Possible transfer of healthcare workers can be done from counties marked by large green bubbles to counties marked by small red bubbles. Note that healthcare workers are but only one major resource in the health system. Treating patients with COVID requires providing (or transferring) materials such as beds, reanimation equipment, and other resources.

## DISCUSSION AND LIMITATIONS

An important confounder was our inability to adjust for the number of importations of COVID-19 in these counties, as more urbanized areas were more likely to have links with countries (via major airports) and other states (especially Northeast states) where the virus could have originated. Even then, we still saw that once an area is seeded with COVID-19, the growth rate was greater in denser areas. Also, there was a difference between stated policy, implementation, and enforcement. Some people might have followed recommended approaches for protective actions, while others might not have complied. These behavioral aspects may also depend on a disconnect between policy messages at different levels of government and exacerbated by rapid updates in a fast-moving pandemic. Therefore, communication management and clarity are of utmost importance during a crisis.

The study was limited to dynamics of the spread. Effects of vaccination, travel bans, and lockdown were not included in the study as part of the model. Unfortunately, there is little published work examining the behavioral aspects of COVID-19 spread and assessing attitudes toward COVID-19 vaccination mandates and the impact of the policies on vaccine uptake and future vaccination behavior. These control measures may have detrimental effects on people’s autonomy, motivation, and willingness to get vaccinated.

## CONCLUSIONS

Population-weighted density can significantly impact the rate of spread of highly contagious diseases. The current COVID-19 pandemic data allowed us to investigate relationships between the population-weighted density and various characteristics of the U.S. population. Our data frame was up-to-date and comprehensive, and our results consistent with previous studies. Our analysis suggests that population-weighted density can be a useful tool to control and manage outbreaks, especially within the early stage of the spread. We used a regression model to study the impact of population-weighted density on COVID-19 spread in the U.S. while controlling for key explanatory variables. We found that population-weighted density was one of the significant predictors of infection rates in the early stage of the disease spread.

People in high-density areas live differently from others. For example, they are more likely to walk or take public transportation in daily life. Due to greater human contact, the disease spreads faster in high population-weighted areas. Therefore, particular attention should be given to prevent spread in the highest-density areas directed by population-weighted density. If the spread is successfully contained early, there will be less stress placed upon the healthcare system, resulting in better healthcare access for those who are sick.

This study highlights the importance of early response and recommends prioritizing public health, especially in densely populated areas, together with investing in robust healthcare systems. The experience with COVID-19 emphasizes the importance of preparedness for future health crises, with investment in research, infrastructure, and pandemic response planning. Public health officials and policymakers may consider the dynamics of the disease presented in the study to design appropriate strategies to control the spread of infectious diseases in densely populated areas.

### Disclosure

The authors report no conflicts of interest.

## Supplementary Material

Online Supplementary Material
