# Supplementary material for: Use of Population Weighted Density Index for Coronavirus Spread in the United States
Source: J Health Econ Outcomes Res. 2024 Jul 17;11(2):1–8. doi: 10.36469/001c.117784 (PMC11259180; doi:10.36469/001c.117784)
Supplement: Online Supplementary Material [file jheor_2024_11_2_117784_237005.pdf]

Journal of Health Economics and Outcomes Research

Online Supplementary Material

Use of Population-Weighted Density Index for Coronavirus Spread in the United States. *JHEOR*. 2024;11(2):1-8. doi:10.36469/jheor.2024.117784

Figures

Figure S1. COVID-19 Spread Model Performance by County 2

Figure S2. Monthly Correlation Coefficient of Weighted Density and Spread 4

Figure S3. NY Monthly Elasticity Trend 5

Figure S4. NY Monthly Intercept Trend 6

Figure S5. CA Monthly Elasticity Trend 7

Figure S6. CA Monthly Intercept Trend 8

Figure S7. P Population-Weighted Density of NY Counties with Respect to the State Average 9

Figure S8. Total Mortality and Total Older Adult Population 10

Figure S9. Relative Mortality and Relative Population of Older Adults (%) 11

Figure S10. Figure S10. COVID-19 Spread Timeline for NY 12

Figure S11. COVID-19 Spread Timeline for CA 13

Figure S12. COVID-19 Spread Timeline for AZ 14

Figure S13. COVID-19 Spread Timeline for IL 15

Tables

Table S1. Correlation Matrix 3

Table S2. Implemented COVID-19 Policies for Selected States 8

Table S3. Population Above and Below the Mean of Explanatory Variables 15

This supplementary material has been provided by the authors to give readers additional information about their work.

This is an open-access article distributed under the terms of the Creative Commons Attribution 4.0 International License (CCBY-4.0). View this license's legal deed at http://creativecommons.org/licenses/by/4.0 and legal code at http://creativecommons.org/licenses/by/4.0/legalcode for more information.

The predicted values of the spread from the model and the actual spread are compared in **Figure S1.** COVID-19 Spread Model Performance by County The model predicts 96% of the variation within the data. The white counties in the map are within the ±5% difference from the actual spread data. Overall, the model captures the dynamics of COVID spread well in the United States. **Figure S1** shows that the counties that had lower spread (eg, Northwest) are slightly overestimated, and the counties that had relative higher spread (eg, Midwest) are slightly underestimated by the model.

Figure S1. COVID-19 Spread Model Performance by County

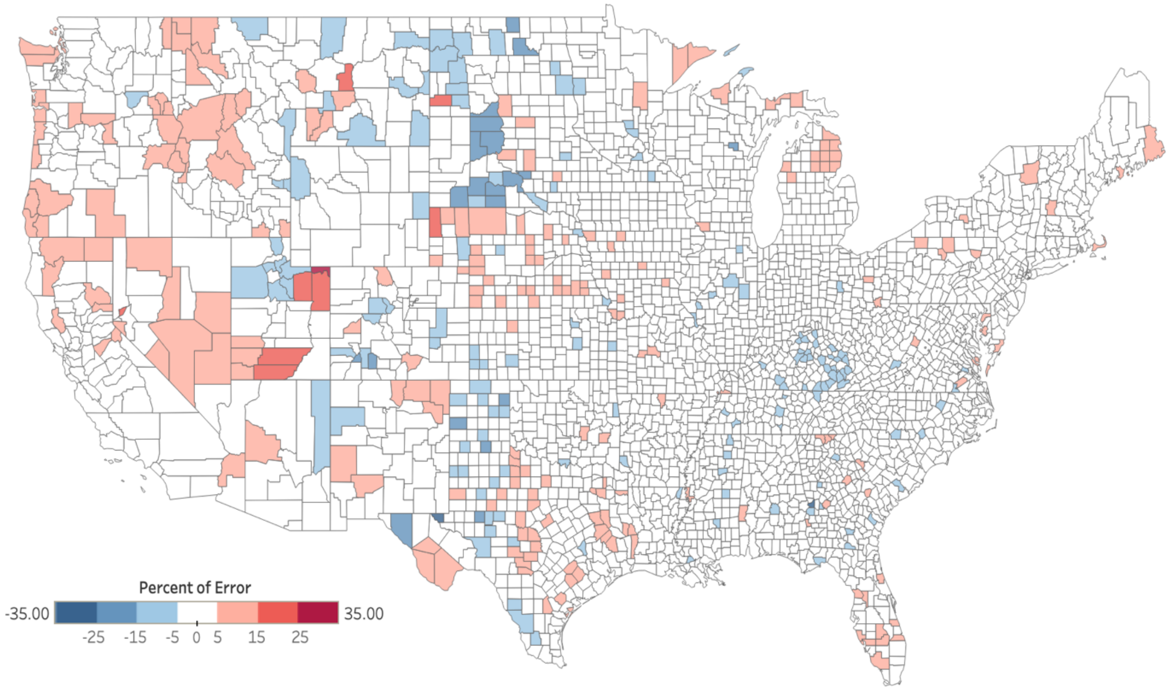

Table S1. Correlation Matrix

	Spread	Mortality	Weighted Density	Seniors	Healthcare Workers	Income	Education	Temperature	Female/Male Ratio
Spread	1								
Mortality	0.955	1							
Weighted density	0.798	0.753	1						
Seniors	0.97	0.9405	0.7639	1					
Healthcare workers	0.924	0.8895	0.8098	0.916	1				
Income	0.135	0.0429	0.1677	0.1849	0.1813	1			
Education	0.421	0.3049	0.4153	0.4853	0.4557	0.6419	1		
Temperature	0.221	0.3072	0.1292	0.1756	0.115	-0.2745	-0.2375	1	
Female/male ratio	0.292	0.3267	0.2253	0.3288	0.3181	0.113	0.2417	0.1801	1

Figure S2. Monthly Correlation Coefficient of Weighted Density and Spread

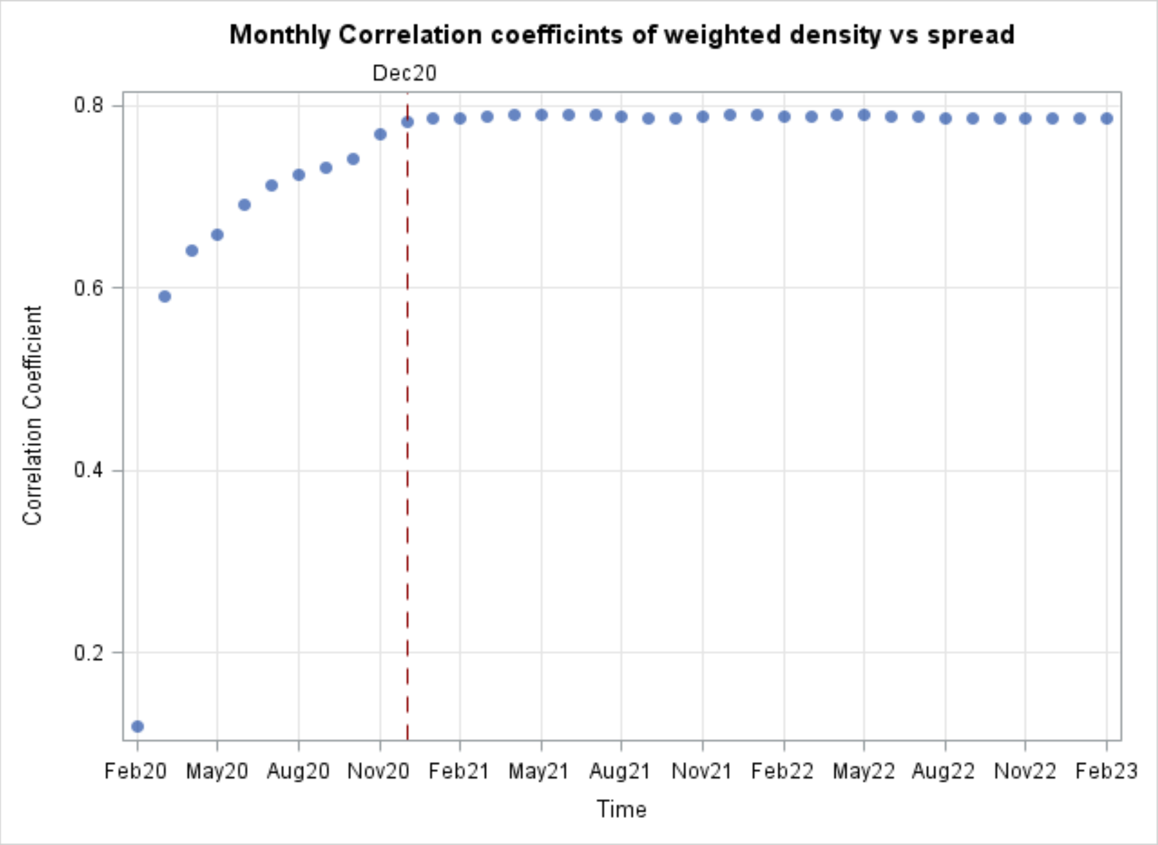

In NY, the maximum rate of spread occurred in March 2020 and declined until January 2021, indicating that NY residents and local administration acted quickly to prevent the spread soon after the outbreak (**Figure S3Figure S3.** NY Monthly Elasticity Trend). While the rate of spread (elasticity) settled around January 2021, the vertical shifts (intercepts) have increased since about March 2022, indicating that NY had a steady increase in COVID-19 cases (**Figure S4**), with a spread rate of nearly zero in about January 2021. Compared with NY, it took about 4 months (until the end of July 2020) to react to the COVID-19 spread in CA (

Figure S5), and the spread rate settled in around November 2020. The final and slightly lower settled rate (than NY) was around February 2022. After the steady rate of increase slope (0.08), the vertical shift in the spread is settled in about March 2022 (

Figure S6), about the same as NY.

Figure S3. NY Monthly Elasticity Trend

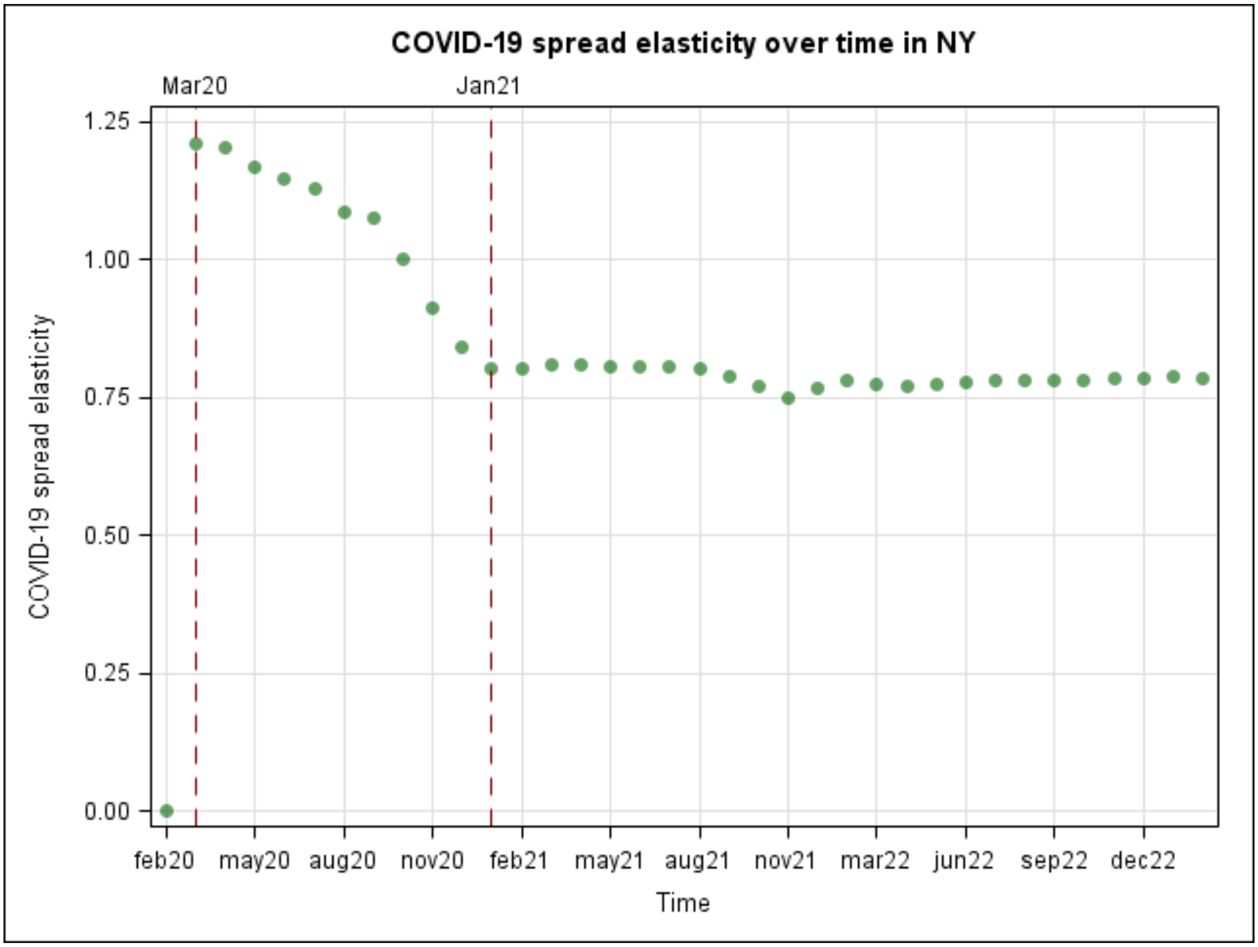

Figure S4. NY Monthly Intercept Trend

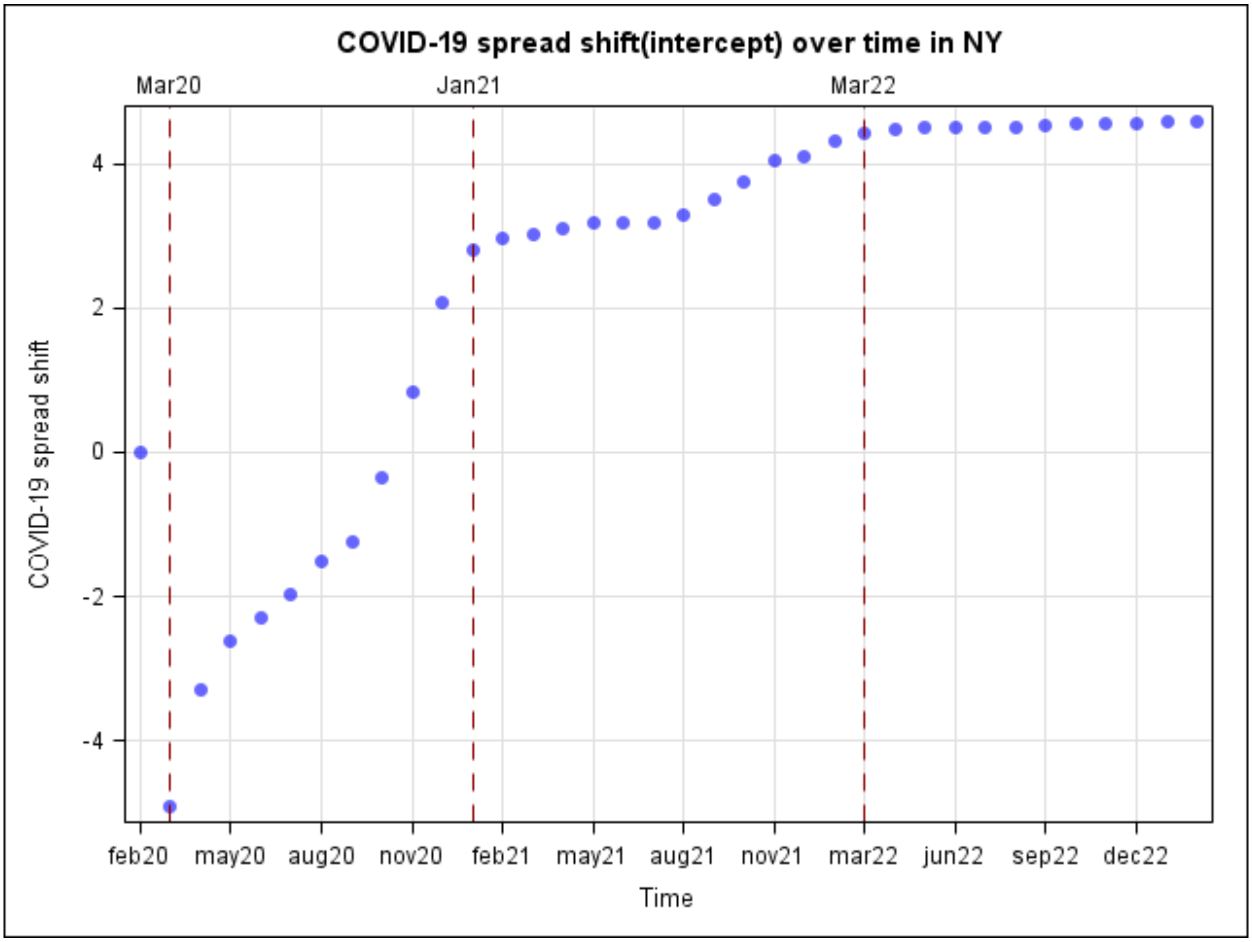

Figure S5. CA Monthly Elasticity Trend

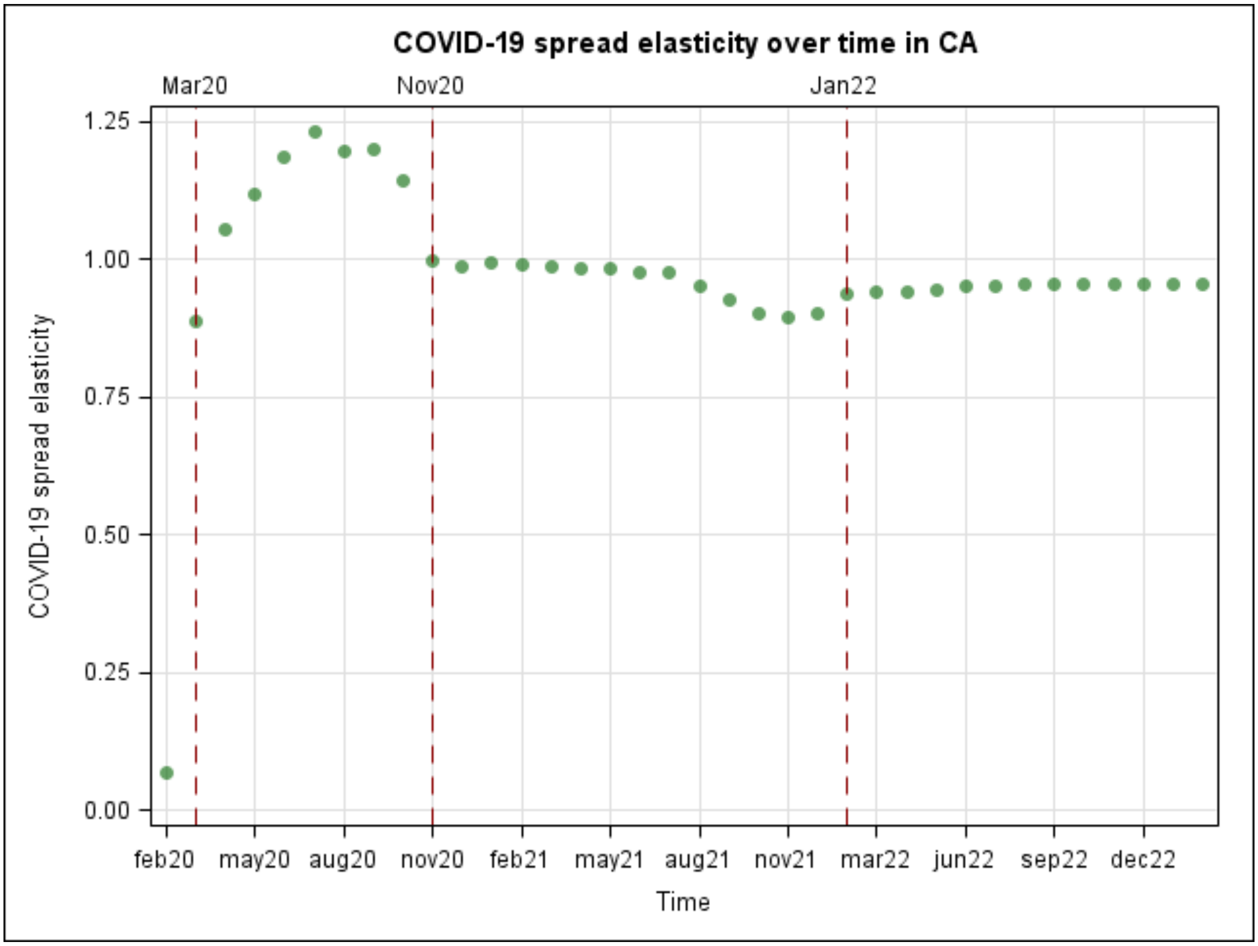

Figure S6. CA Monthly Intercept Trend

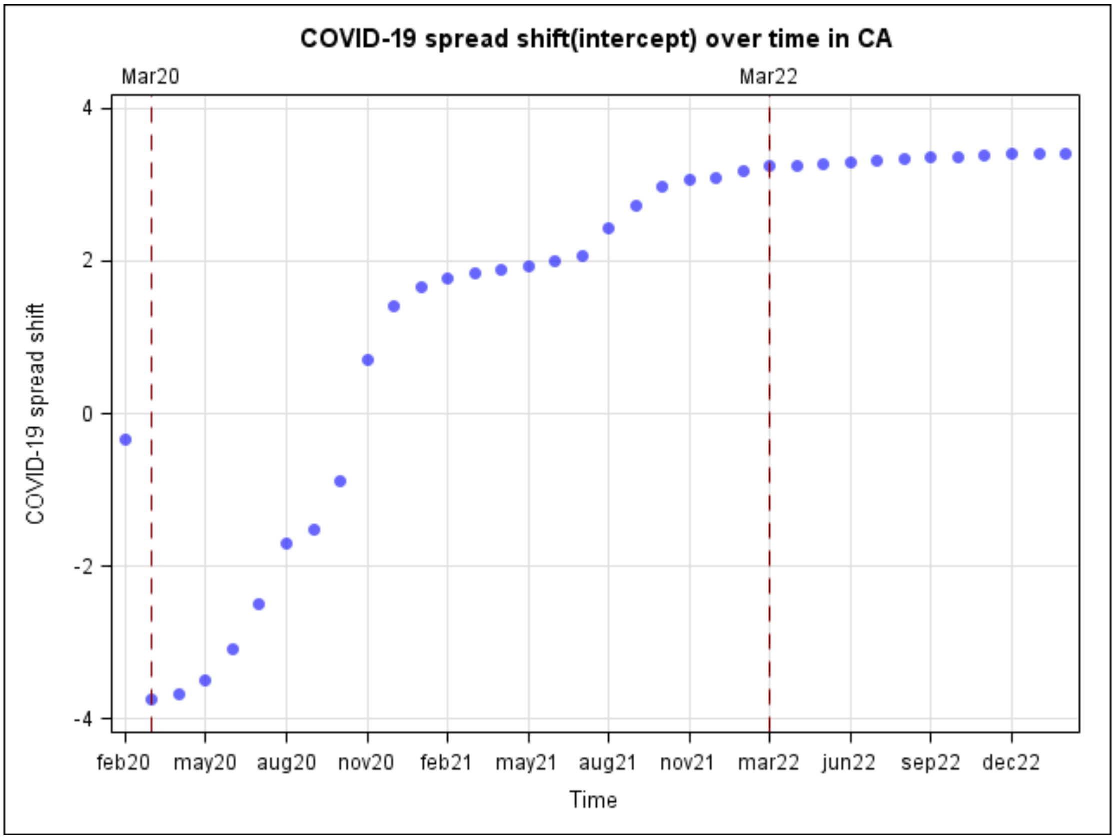

Table S2. Implemented COVID-19 Policies for Selected States

States	Date Started (M/D/Y)	COVID-19 Policy	Date Ended (M/D/Y)
AZ	4/7/20	Travel advisory	5/12/20
AZ	3/31/20	Lockdown	5/15/20
AZ	12/28/20	Vaccine	—
CA	11/13/20	Travel advisory	6/15/21
CA	6/18/20	Mask	3/1/21
CA	3/19/20	Lockdown	5/27/20
CA	12/14/20	Vaccine	—
FL	3/24/20	Travel advisory	8/6/20
FL	3/16/20	Lockdown	6/3/20
FL	1/19/21	Vaccine	—
IL	7/6/20	Travel advisory	9/7/21
IL	5/1/20	Mask	6/11/21
IL	8/30/21	Mask	2/28/22
IL	3/21/20	Lockdown	5/29/20

States	Date Started (M/D/Y)	COVID-19 Policy	Date Ended (M/D/Y)
IL	1/25/21	Vaccine	—
NY	6/25/20	Travel advisory	4/1/21
NY	4/15/20	Mask	1/25/22
NY	1/25/22	Mask	2/10/22
NY	3/20/20	Lockdown	6/27/20
NY	12/14/20	Vaccine	—
TX	3/26/20	Travel advisory	5/21/20
TX	7/3/20	Mask	3/10/21
TX	3/23/20	Lockdown	5/6/20
TX	3/11/21	Vaccine	—

Figure S7. Population-Weighted Density of NY Counties with Respect to the State Average

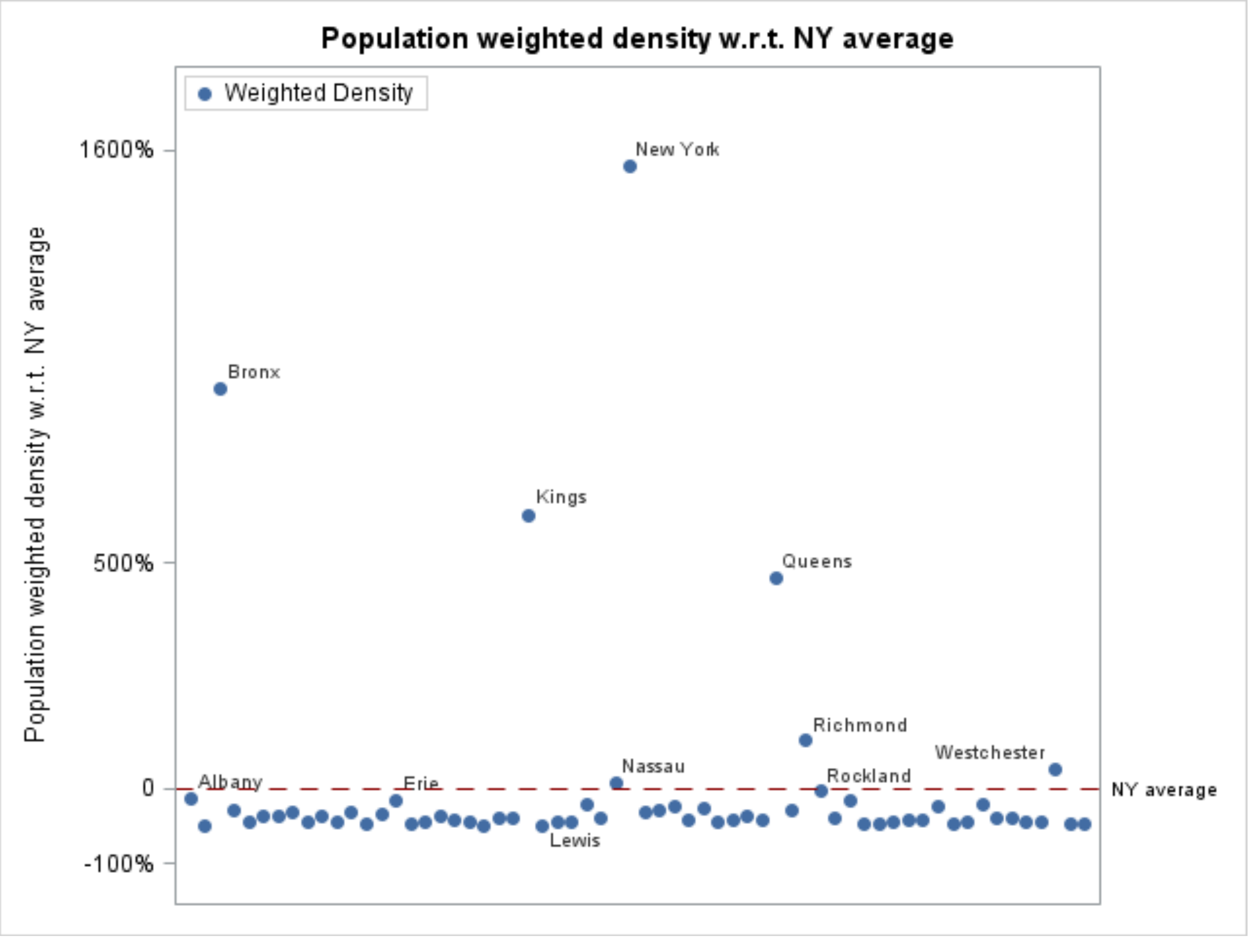

Figure S8. Total Mortality and Total Older Adult Population

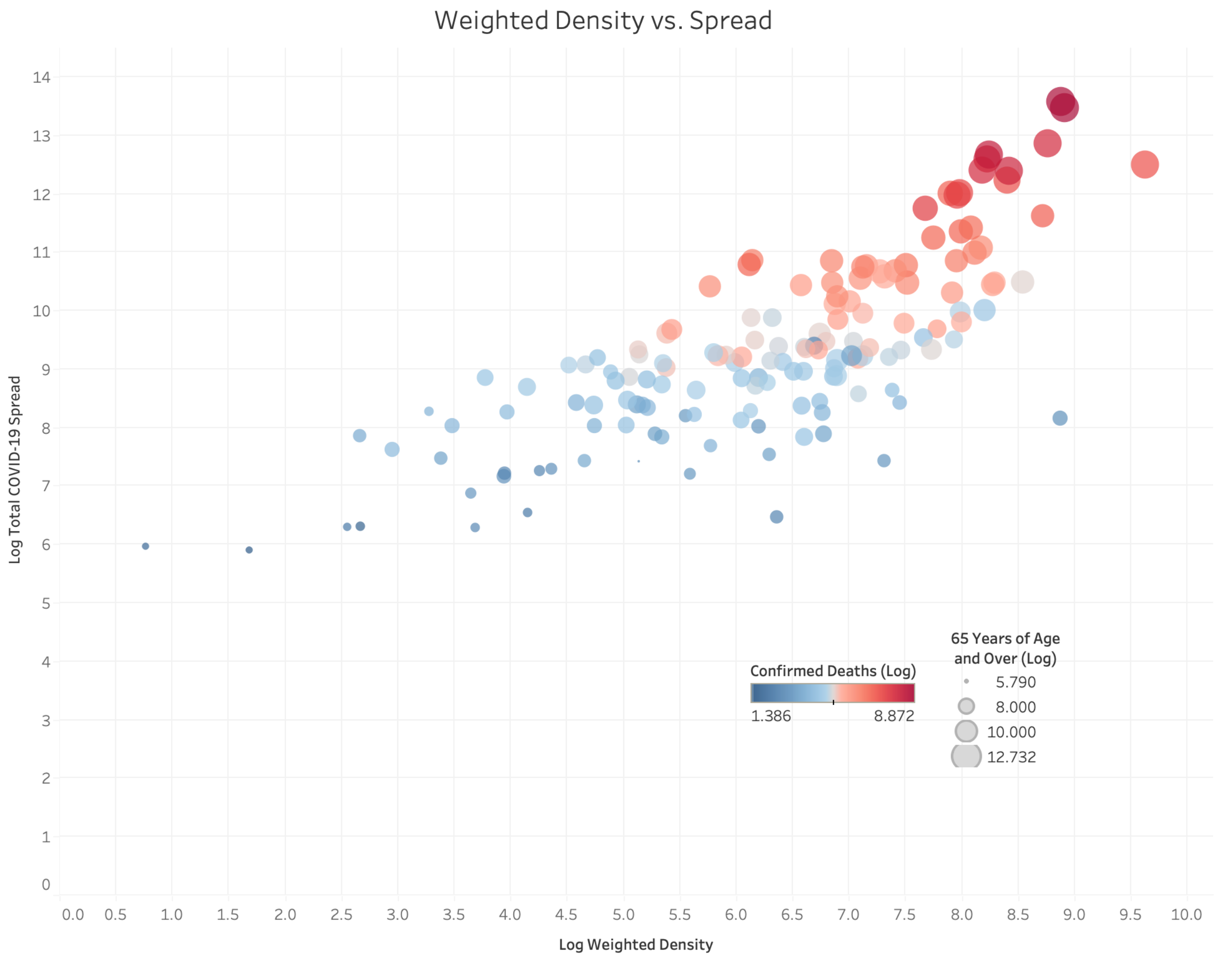

Figure S9. Relative Mortality and Relative Population of Older Adults (%)

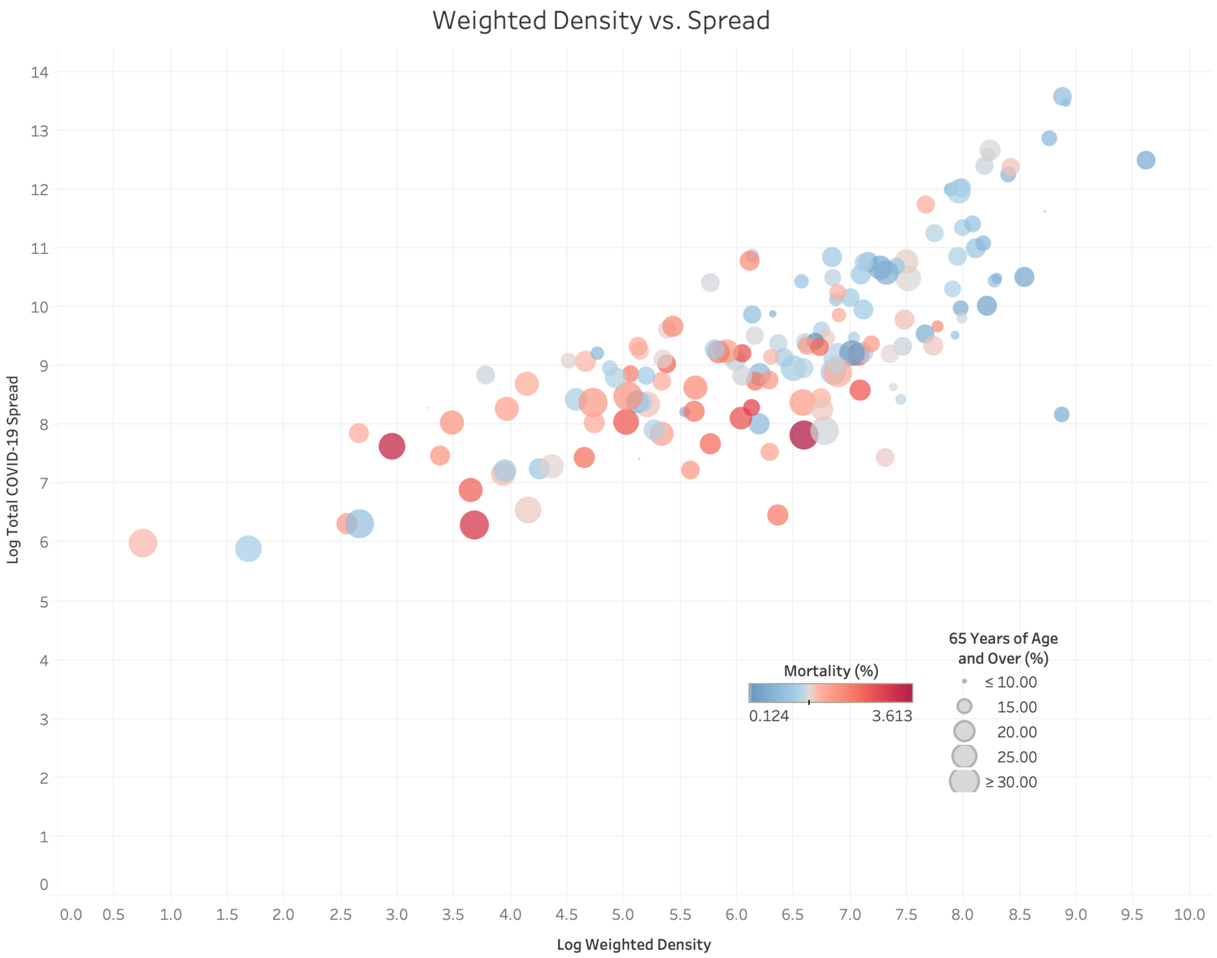

Heavily dense counties have more older adults, who have greater mortality (**Figure S8)**. However, if we consider the relative senior population vs relative mortality (**Figure S9**), we observe relatively high mortality and senior population in moderately populated counties. The overall shape of spread among older adults aligns with the national spread, which explains the 97% correlation.

Figure S10. COVID-19 Spread Timeline for NY

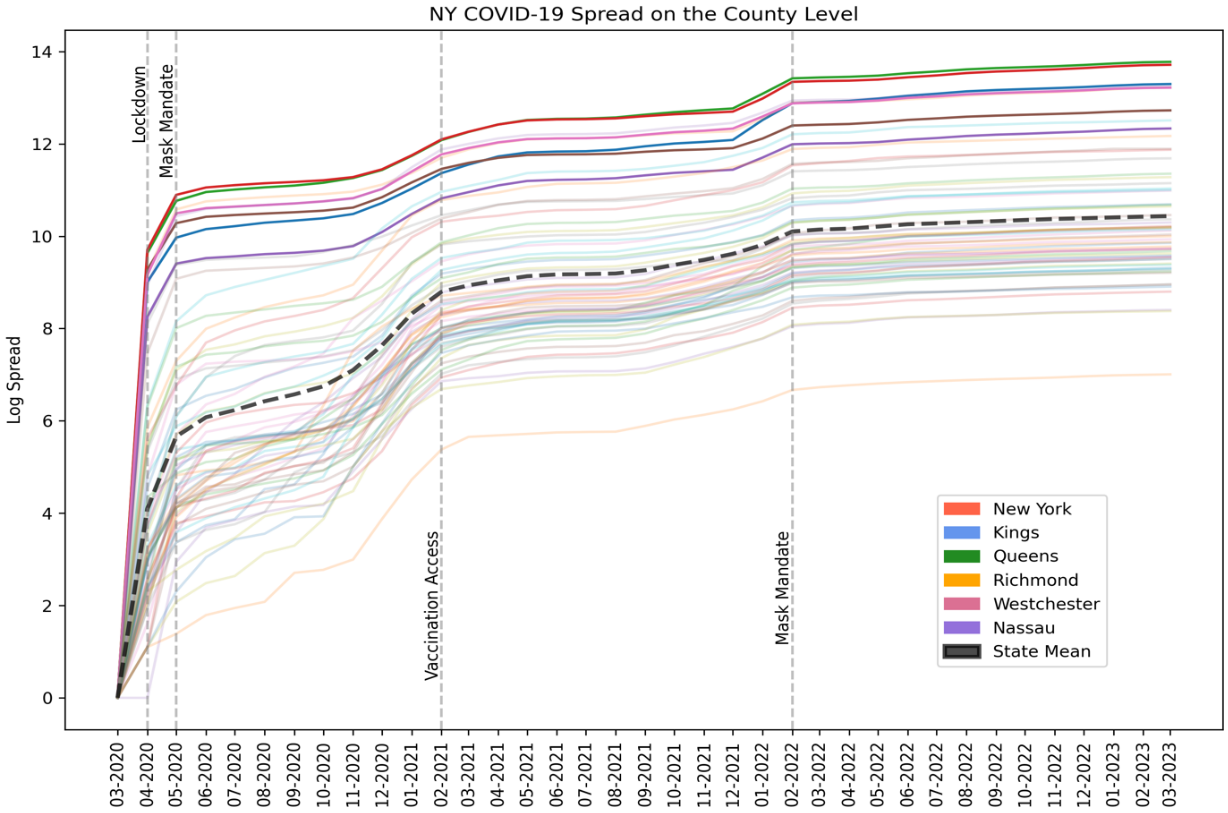

Figure S11. COVID-19 Spread Timeline for CA

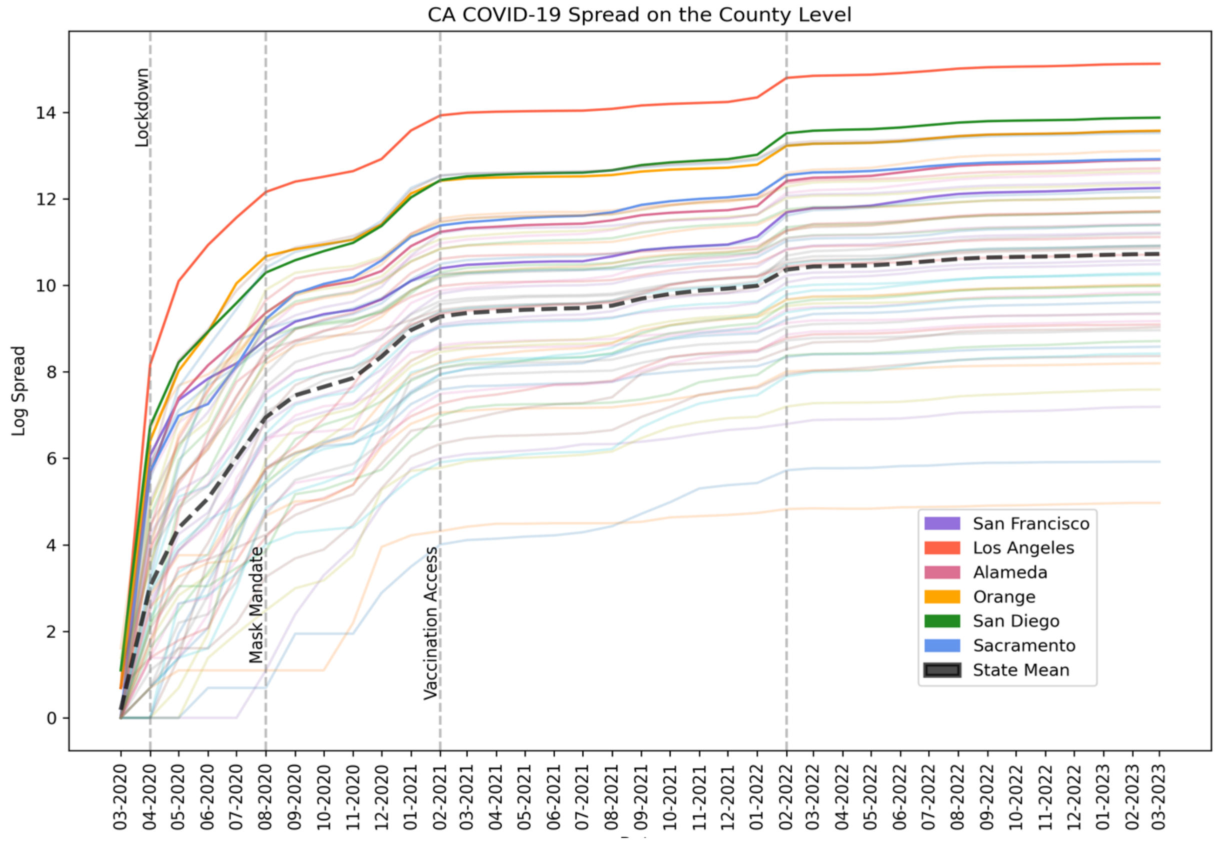

Figure S12. COVID-19 Spread Timeline for AZ

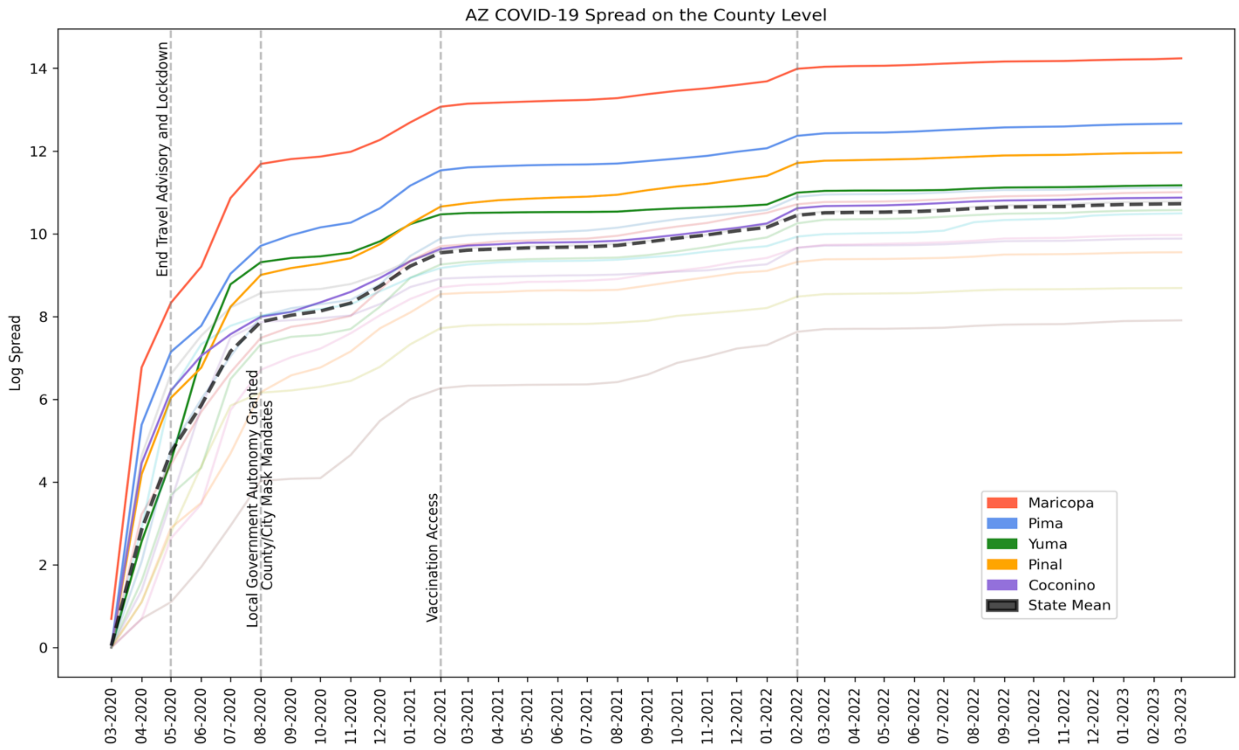

Figure S13. COVID-19 Spread Timeline for IL

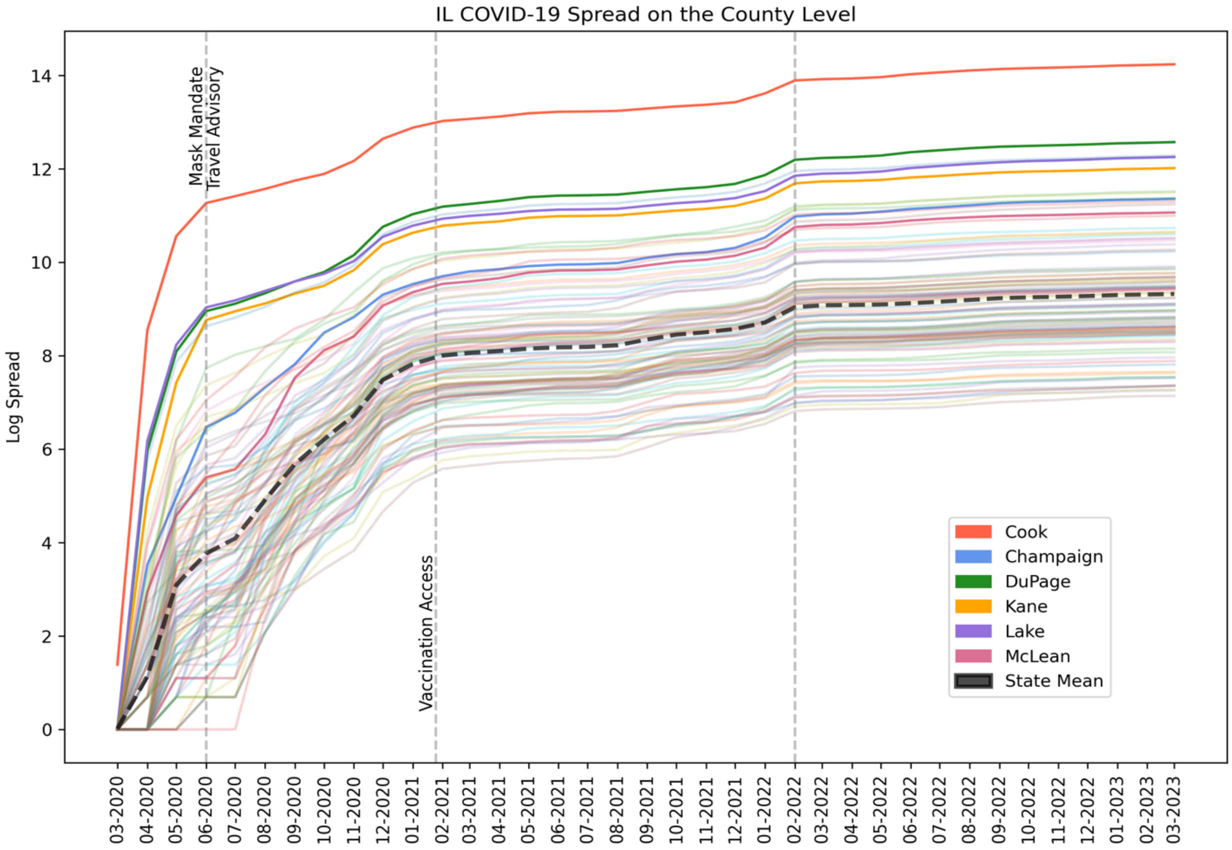

Table S3. Population Above and Below the Mean of Explanatory Variables

Variables	Mean	Population Above the Mean	Population Below the Mean	Percentage Above the Mean	Percentage Below the Mean
Weighted density	1262.38	253669477	64888685	79.63	20.37
Temperature	54.05	199674571	118883591	62.68	37.32
Education level	23.02	253057141	65501021	79.44	20.56
Healthcare workers	6545.48	241331168	77226994	75.76	24.24
Seniors (age $\geq$ 65)	17773.75	251350957	67207205	78.9	21.1
Income per capita	53326.34	218210071	100348091	68.5	31.5
Female/male ratio	98.62	266686171	51871991	83.72	16.28
